# Ambient-pressure hydrogenation of CO_2_ into long-chain olefins

**DOI:** 10.1038/s41467-022-29971-5

**Published:** 2022-05-03

**Authors:** Zhongling Li, Wenlong Wu, Menglin Wang, Yanan Wang, Xinlong Ma, Lei Luo, Yue Chen, Kaiyuan Fan, Yang Pan, Hongliang Li, Jie Zeng

**Affiliations:** 1 Hefei National Research Center for Physical Sciences at the Microscale, Key Laboratory of Strongly-Coupled Quantum Matter Physics of Chinese Academy of Sciences, National Synchrotron Radiation Laboratory, Key Laboratory of Surface and Interface Chemistry and Energy Catalysis of Anhui Higher Education Institutes, Department of Chemical Physics, University of Science and Technology of China, Hefei, Anhui 230026 PR China; 2 Songshan Lake Materials Laboratory, Dongguan, Guangdong 523808 PR China; 3 Beijing National Laboratory for Condensed Matter Physics and Institute of Physics, Chinese Academy of Sciences, Beijing, 100190 PR China

**Keywords:** Heterogeneous catalysis, Catalytic mechanisms, Chemical engineering

## Abstract

The conversion of CO_2_ by renewable power-generated hydrogen is a promising approach to a sustainable production of long-chain olefins (C_4+_^=^) which are currently produced from petroleum resources. The decentralized small-scale electrolysis for hydrogen generation requires the operation of CO_2_ hydrogenation in ambient-pressure units to match the manufacturing scales and flexible on-demand production. Herein, we report a Cu-Fe catalyst which is operated under ambient pressure with comparable C_4+_^=^ selectivity (66.9%) to that of the state-of-the-art catalysts (66.8%) optimized under high pressure (35 bar). The catalyst is composed of copper, iron oxides, and iron carbides. Iron oxides enable reverse-water-gas-shift to produce CO. The synergy of carbide path over iron carbides and CO insertion path over interfacial sites between copper and iron carbides leads to efficient C-C coupling into C_4+_^=^. This work contributes to the development of small-scale low-pressure devices for CO_2_ hydrogenation compatible with sustainable hydrogen production.

## Abstract (web summary)

The conversion of CO2 by renewable power-generated hydrogen is a promising approach to a sustainable production of long-chain olefins. Here the authors report a Cu-Fe catalyst which achieves the hydrogenation of CO2 into long-chain olefins under ambient pressure via the synergy of carbide mechanism and CO insertion mechanism.

## Introduction

As the culprit for greenhouse effect, CO_2_, especially those in high purity released from cement manufacturing, breweries, and fuel processing facilities, can be regarded as a promising candidate to synthesize chemicals which are currently produced from fossil resources. Long-chain olefins (C_4+_^=^) are versatile industrial feedstocks for a variety of value-added products such as synthetic lubricants, high-octane gasoline, biodegradable detergents, new polymers, agricultural chemicals, coatings, and corrosion inhibitors^1,2^. The prevalent method for the synthesis of these olefins is based on oligomerization of ethylene which is mostly produced from petroleum resources^3,4^. The use of C_4+_^=^ as industrial feedstocks would play a pivotal role in the development of a sustainable society if C_4+_^=^ could be directly obtained from CO_2_ hydrogenation. To ensure the whole process carbon negative, H_2_ must be produced from water electrolysis powered by renewable energy instead of coal gasification or reforming of natural gas^5,6^. Considering that electrolysis is distributed and produced in small-scale devices, it would be attractive to perform the subsequent CO_2_ hydrogenation in ambient-pressure units for matching the manufacturing scales and flexible on-demand production^7,8^.

For CO_2_ hydrogenation, ambient pressure is adverse to the formation of liquid long-chain olefins based on Le Chatelier’s principle. Currently, olefins produced from CO_2_ hydrogenation are mainly in the gaseous range of C_2-4_^=^, where the corresponding catalysts generally comprised metal oxides for methanol synthesis and zeolites for methanol-to-olefin process^9–11^. Limited catalysts targeting on long-chain olefins were operated under high pressure^12–14^. For instance, an iron aluminum oxide exhibited high selectivity (66.8%) for long-chain olefins (C_4+_^=^) under 35 bar^15^. However, it is not simply a case of lowering the pressure for these catalysts if one intends to achieve ambient-pressure synthesis of long-chain olefins.

Designing a tandem process including CO-intermediate and methanol-intermediate routes represents a successful strategy for CO_2_ hydrogenation into long-chain products^16–18^. To carter to the ambient-pressure condition, we should choose CO-intermediate route because low-pressure benefits reverse-water-gas-shift (RWGS) reaction but suppresses methanol synthesis process^8^. In this regard, the challenge lies in seeking active sites for Fischer-Tropsch synthesis (FTS) under ambient pressure. C–C coupling during FTS generally includes carbide mechanism and CO insertion mechanism^19–22^. The carbide mechanism involves the dissociation of CO into surface carbon, the hydrogenation of surface carbon into CH_x_* (*x* = 1, 2, or 3) intermediates, the surface polymerization of CH_x_* (chain growth), and the hydrogenation (chain termination) to form hydrocarbon products^19^. The low total pressure induces low surface coverage of CH_x_* due to the insufficient dissociation of CO, resulting in the short-chain length and a large amount of undissociated CO molecules. If these undissociated CO molecules are utilized as the monomer unit for chain growth, namely CO insertion mechanism^21^, it is promising to achieve the formation of long-chain products under ambient pressure.

Herein, we report a Cu–Fe catalyst which was operated under ambient pressure with comparable C_4+_^=^ selectivity to that of the state-of-the-art catalysts optimized under high pressure toward CO_2_ hydrogenation (Supplementary Fig. 1 and Supplementary Table 1). The catalyst activated from delafossite oxides CuFeO_2_ contained Cu, iron oxides, and iron carbides, which was denoted as activated CuFeO_2_. Iron oxides enabled RWGS reaction to produce CO, while CO underwent carbide mechanism over iron carbides and experienced CO insertion over interfacial sites between copper and iron carbides. The synergy of carbide path and CO insertion path resulted in a high C_4+_^=^ selectivity of 66.9% (excluding CO) and a CO_2_ conversion of 27.3% under 1 bar (H_2_:CO_2_ = 3:1) with a space velocity of 2400 mL h^−1^ g_cat_^−1^ at 320 °C. Moreover, we found that increasing the total pressure (30 bar) was able to refresh the deactivated catalyst during CO_2_ hydrogenation under ambient pressure.

## Results and discussion

### Catalytic properties

**Fig. 1:**
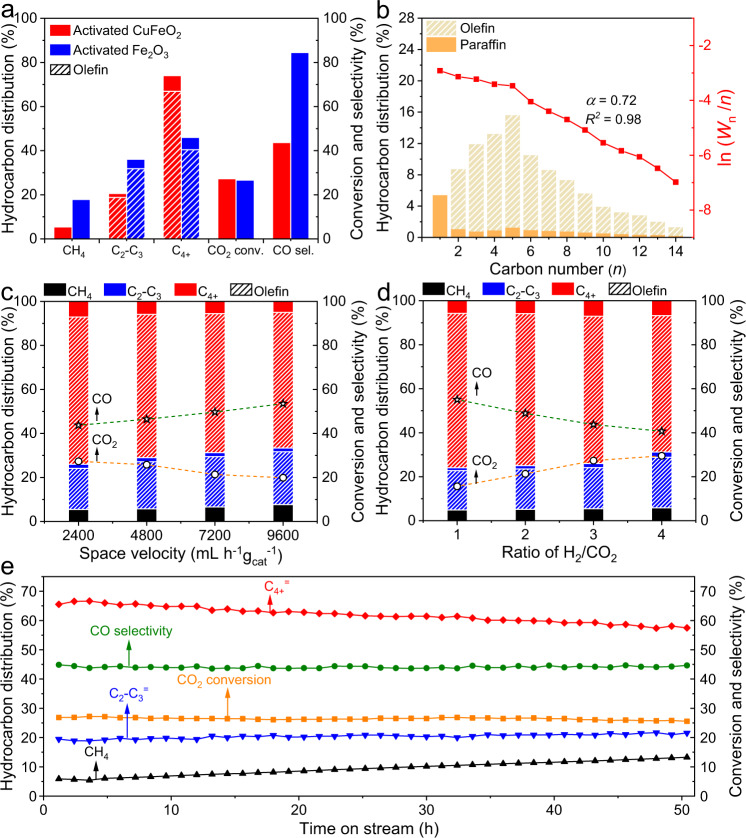
Catalytic properties toward CO_2_ hydrogenation. **a** The hydrocarbon product distribution, CO_2_ conversion, and CO selectivity of activated CuFeO_2_ and activated Fe_2_O_3_. **b** The detailed hydrocarbon product distribution, the ASF plot, and the corresponding *α* value of activated CuFeO_2_. *α* is the probability of chain growth. *R*^*2*^ is the coefficient of determination, describing the goodness of linear fitting. *W*_n_ is the weight fraction of a product with *n* carbon atoms. CO_2_ conversion and product selectivity at **c** different space velocities and **d** different H_2_:CO_2_ ratios over activated CuFeO_2_. **e** Stability test of activated CuFeO_2_. For panels **a** and **b**, the reaction was conducted under 1 bar (H_2_:CO_2_ = 3:1) with a space velocity of 2400 mL h^−1^ g_cat_^−1^ at 320 °C after 4 h on stream. The reaction conditions for panels **c**–**e** are similar to that for panel **a** except for the space velocity, H_2_:CO_2_ ratio, and time on stream, respectively. The selectivity for hydrocarbon product excludes CO.

We prepared the catalysts via a hydrothermal method. The resulting sample exhibited a typical delafossite-type structure with homogeneous distribution of Cu, Fe, and O elements, which was denoted as fresh CuFeO_2_ (Supplementary Figs. 2 and 3). The fresh CuFeO_2_ was activated via H_2_ reduction under 4 bar at 400 °C for 2 h, followed by being exposed to 1 bar of mixed gas (H_2_:CO_2_ = 3:1) with a space velocity of 2,400 mL h^−1^ g_cat_^−1^ at 320 °C for 4 h since when the conversion of CO_2_ became stable. The obtained catalyst was designated as activated CuFeO_2_. The catalytic properties of activated CuFeO_2_ were evaluated in a fixed-bed reactor under 1 bar (H_2_:CO_2_ = 3:1) with a space velocity of 2,400 mL h^−1^ g_cat_^−1^ at 320 °C for 4 h on stream. The ambient pressure inevitably resulted in high CO selectivity of 43.7%. The selectivity for C_4+_ hydrocarbons was 74.0% (excluding CO) with an extremely high ratio (9.4) of olefin to paraffin (o/p ratio) at a CO_2_ conversion of 27.3% (Fig. 1a and Supplementary Table 1). Though activated CuFeO_2_ was operated under ambient pressure, the C_4+_^=^ selectivity was as high as 66.9%, which was comparable to that over the state-of-the-art catalysts optimized for high-pressure conversion (Supplementary Table 1). The CH_4_ selectivity reached 5.4%, while the selectivity for C_2-3_ hydrocarbons was 20.6%. Figure 1b shows the distribution of hydrocarbon products, well matching the Anderson–Schulz–Flory (ASF) distribution. The probability of chain growth (*α*) was calculated as 0.72, while the coefficient of determination (*R*^2^) was 0.98. As indicated by the high linearity and high CO selectivity, the reaction tandemly proceeds along a RWGS reaction and a typical FTS process obeying the ASF model. Once following ASF distribution, the carbon chain grew via one-carbon-atom process while the *α* value was independent on carbon numbers^23^. Notably, the hydrocarbon distribution of activated CuFeO_2_ behaved differently from that of a similar reported catalyst even under the same condition^24^ (Supplementary Table 2 and Supplementary Fig. 4). In addition, the pre-reduction in 1 bar of H_2_ did no obviously influence the catalytic activity and selectivity compared with the catalyst pre-reduced in 4 bar of H_2_ (Supplementary Table 3).

By comparison, Fe_2_O_3_ after the same activation procedure (denoted as activated Fe_2_O_3_) was tested under 1 bar (H_2_:CO_2_ = 3:1) with a space velocity of 2,400 mL h^−1^ g_cat_^−1^ at 320 °C for 4 h on stream. The C_4+_ selectivity reached 46.0% (excluding CO) with an o/p ratio of 7.2 at a CO_2_ conversion of 26.6%, whereas the selectivities for CH_4_ and C_2-3_ hydrocarbons were 17.9% and 36.1%, respectively (Fig. 1a and Supplementary Table 1). The C_4+_^=^ selectivity was 40.4%, lower than that (66.9%) over activated CuFeO_2_. As shown in Supplementary Figure 5, the distribution of hydrocarbon products for activated Fe_2_O_3_ also followed the ASF distribution with an *α* value of 0.59 and a high *R*^2^ value of 0.99. When the total pressure increased to 30 bar, the C_4+_^=^ selectivity increased to 50.2% (Supplementary Table 1). In addition, we prepared ZnO-ZrO_2_ solid solution supported on a Zn-modified SAPO-34 zeolite (ZnZrO/SAPO) as a reference catalyst. This catalyst was reported as a highly selective catalyst toward light olefins under high pressure^25^. Considering that ZnZrO/SAPO was active at high temperature (360–400 °C), we tested its catalytic performance under 1 bar (H_2_:CO_2_ = 3:1) with a space velocity of 2,400 mL h^−1^ g_cat_^−1^ at 380 °C. Under this condition, the major product was light olefins (C_2_–C_4_^=^) with the selectivity of 61.4% instead of long-chain olefins (C_4+_^=^) with the selectivity of 3.5% (Supplementary Table 1). Moreover, the selectivities for CO and methane (excluding CO) boosted to 85.1% and 30.3%, respectively (Supplementary Table 1), because lowering the pressure suppresses methanol synthesis due to Le Chatelier’s principle.

To investigate the robustness of activated CuFeO_2_, we varied the space velocity and the ratio of H_2_ to CO_2_ over activated CuFeO_2_. When the space velocity increased from 2400 to 9600 mL h^−1^ g_cat_^−1^, the C_4+_^=^ selectivity decreased slightly from 66.9% to 61.6% (Fig. 1c). Moreover, the selectivity for CH_4_, C_2-3_, and C_4+_ along with o/p ratios also exhibited slight variations, while the conversion of CO_2_ decreased from 27.3% to 19.8% (Fig. 1c). When the ratio of H_2_ to CO_2_ increased from 1 to 4 with the space velocity of 2,400 mL h^−1^ g_cat_^−1^, the C_4+_^=^ selectivity dropped from 70.2% to 62.0%, while the conversion of CO_2_ rose from 15.6% to 29.5% (Fig. 1d). Therefore, the selectivity for long-chain olefins is insensitive to both the space velocity and the ratio of H_2_ to CO_2_, indicating high robustness of activated CuFeO_2_ which applied to a wide range of reaction conditions.

We further investigated the stability of activated CuFeO_2_ under 1 bar (H_2_:CO_2_ = 3:1) with a space velocity of 2,400 mL h^−1^ g_cat_^−1^ at 320 °C. After 50 h on stream, the conversion of CO_2_ kept stable, whereas the selectivity for long-chain olefins (C_4+_^=^), unfortunately, dropped to 57.6% (Fig. 1e). Meanwhile, the selectivity for methane increased to 14.6%. Thus, activated CuFeO_2_ was not stable enough during ambient-pressure hydrogenation of CO_2_.

### Regeneration of activated CuFeO_2_

**Fig. 2:**
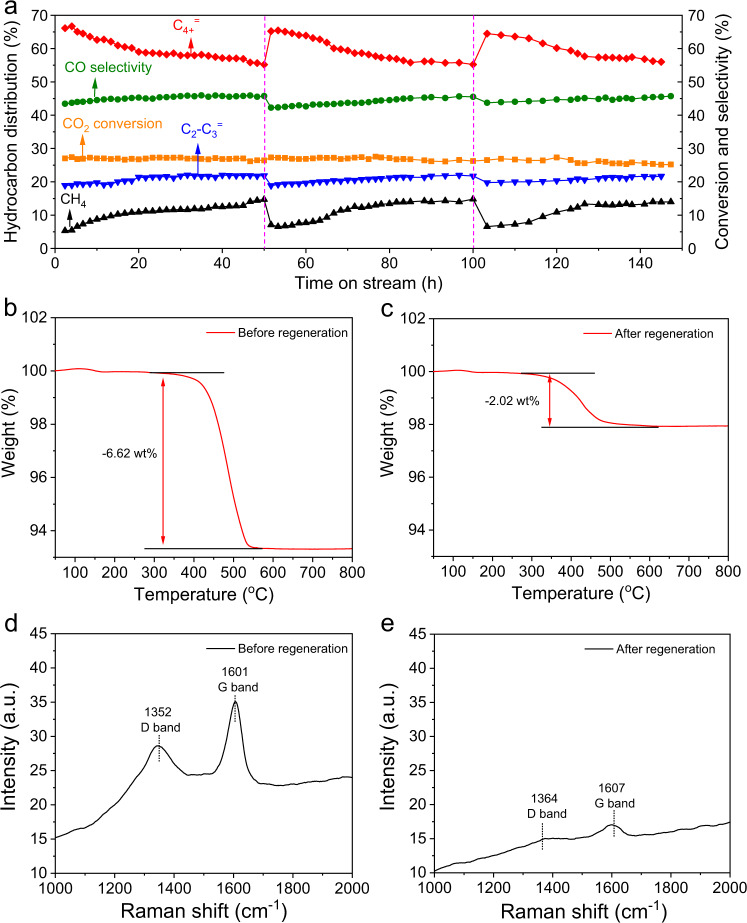
Regeneration of activated CuFeO_2_. **a** Long-term stability test with regeneration over activated CuFeO_2_. The reaction was conducted under 1 bar (H_2_:CO_2_ = 3:1) with a space velocity of 2400 mL h^−1^ g_cat_^−1^ at 320 °C. The purple dash vertical lines refer to the regeneration treatment that was conducted under 30 bar (H_2_:CO_2_ = 3:1) with a space velocity of 2400 mL h^−1^ g_cat_^−1^ at 320 °C for 4 h on stream. TGA profiles of activated CuFeO_2_ **b** before regeneration treatment and **c** after regeneration treatment. Raman spectra of activated CuFeO_2_ **d** before regeneration treatment and **e** after regeneration treatment.

We notice that an elevated total pressure (30 bar) with high H_2_:CO_2_ ratio (3:1) enables the coverage of surface hydrogen on catalysts to become high enough for minimizing carbon formation^26,27^. Inspired by these points, we propose that increasing the total pressure serves as a promising approach to the regeneration of the catalysts. When the reaction proceeded under 1 bar at 320 °C for 50 h on stream, the C_4+_^=^ selectivity declined to 55.2% (Fig. 2a). Afterward, the total pressure was increased to 30 bar. Under this condition, the C_4+_^=^ selectivity reached 66.3% under 30 bar, almost equal to that (66.9%) under ambient pressure (Supplementary Table 3). Compared with the results obtained under 1 bar, the major difference lies in the significant decrease of CO selectivity (12.3%) and the appearance of oxygenated products with the selectivity of 7.5% (Supplementary Table 3). Based on Le Chatelier’s principle, the elevated pressure promotes the conversion of CO to hydrocarbons and oxygenated products, while RWGS is insensitive to pressure, accounting for the decreased selectivity for CO. The appearance of oxygenated products indicates the existence of CO insertion mechanism in the catalytic system^21^. After operating the catalyst under 30 bar for 4 h, we lowered the total pressure back to 1 bar. It is worth noting that the C_4+_^=^ selectivity recovered to 65.2% (Fig. 2a). When we repeated the regeneration procedure, activated CuFeO_2_ resumed its high selectivity for C_4+_^=^ every time. Therefore, elevating the total pressure represents a convenient regeneration method which does not need to switch gases or unload the catalysts from the reactor.

The effect of the regeneration was investigated by means of thermogravimetric analysis (TGA) in N_2_ atmosphere. For activated CuFeO_2_ that had been operated under 1 bar for 50 h (denoted as the catalyst before regeneration), the weight was lost by 6.62 wt% (Fig. 2b). The catalyst after working under 1 bar for 50 h and subsequently under 32 bar for 4 h was denoted as the catalyst after regeneration. During TGA tests, activated CuFeO_2_ after regeneration lost 2.02 wt% weight, which was lower than that (6.62 wt%) before regeneration (Fig. 2c). As such, high-pressure treatment cleaned long-chain hydrocarbons to refresh the catalyst. We further conducted Raman measurements. The peaks at 1352 and 1364 cm^−1^ were assigned to disordered carbon (D band), while those at 1601 and 1607 cm^−1^ corresponded to graphite (G band) (Fig. 2d, e)^28^. The intensities of these two peaks for activated CuFeO_2_ before regeneration were higher than those for the catalyst after regeneration (Fig. 2d, e). In this case, high-pressure treatment is also able to remove disordered carbon and graphite.

In addition, we conducted Brunauer–Emmett–Teller (BET) measurements of activated CuFeO_2_ after different treatments. For clarity, we denote samples I, II, III, and IV as the activated CuFeO_2_ after reaction for 0, 10, 20 h, and regeneration, respectively. The BET surface areas of samples I, II, III, and IV were measured as 2.88, 2.62, 2.35, and 2.96 m^2^ g^−1^, respectively (Supplementary Fig. 6). In this case, the BET surface area decreased with the prolonged reaction time and recovered after regeneration. However, the variation in surface area of the samples after different treatments was not prominent, presumably consistent with the stable conversion of CO_2_. There must be other underlying mechanisms for catalyst deactivation and regeneration in addition to carbon deposition. To this end, we conducted energy dispersive X-ray (EDX) elemental mapping characterizations of samples I, III, and IV. We found that the spatial overlap between Cu and Fe elements of sample III is obviously smaller than that of sample I and that of sample IV (Supplementary Figs. 7–9). As such, we speculate that the deactivation and regeneration are presumably associated with the segregation and re-dispersion of Cu and Fe elements.

### Structural and electronic characterizations of activated CuFeO_2_

**Fig. 3:**
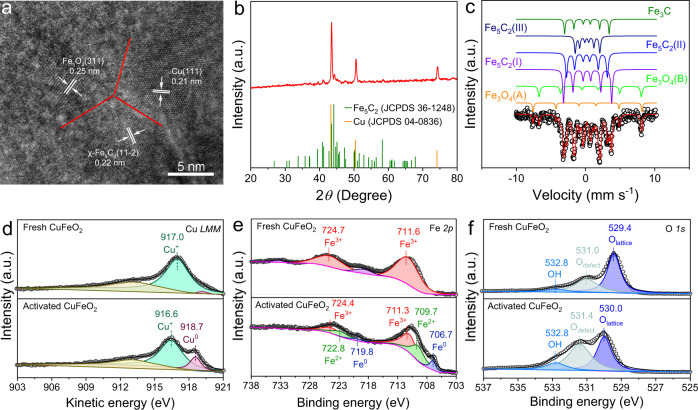
Structural characterizations of activated CuFeO_2_. **a** HAADF image of activated CuFeO_2_. **b** XRD profiles of activated CuFeO_2_. **c** Mössbauer spectra of activated CuFeO_2_. **d** Cu *LMM* Auger, **e** Fe 2*p* XPS, and **f** O 1*s* XPS spectra of fresh and activated CuFeO_2_.

To explore the nature of active sites, we resorted to multiple structural characterizations. Comparing scanning electron microscopy (SEM) images of fresh and activated CuFeO_2_, we found that the activation procedure etched the initial polyhedral particles into a porous structure (Supplementary Figs. 2a and 10). Figure 3a shows a high-angle annular dark-field scanning transmission electron microscopy (HAADF-STEM) image of activated CuFeO_2_. The lattice parameters of 0.21, 0.25, and 0.22 nm were ascribed to Cu(111), Fe_3_O_4_(311), and χ-Fe_5_C_2_(11-2) facets, respectively. The interfaces between these facets marked by red lines were clearly identified. Supplementary Figure 7a shows EDX elemental mapping images, suggesting that activated CuFeO_2_ comprised Cu, Fe, O, and C elements. In comparison with the elemental mapping images of fresh CuFeO_2_, the compositional line profile of activated CuFeO_2_ indicated that the homogeneously distributed Cu and Fe elements underwent obvious segregation after the activation (Supplementary Fig. 7b). Moreover, the activation treatment led to intimate contact between Cu and Fe species as indicated by elemental mapping images and compositional line profile (Supplementary Fig. 7). The phase segregation was further verified by the X-ray diffraction (XRD) profile of activated CuFeO_2_. As shown in Fig. 3b, the characteristic XRD peaks were assigned to the phases of Hägg carbides (*χ*-Fe_5_C_2_) and pure Cu. The Mössbauer spectra of activated CuFeO_2_ showed that iron phases include χ-Fe_5_C_2_, Fe_3_C, and Fe_3_O_4_. Specially, χ-Fe_5_C_2_, Fe_3_C, and Fe_3_O_4_ occupied 73.6%, 9.8%, and 16.6% of the total iron phases (Fig. 3c and Supplementary Table 4). The iron phases of activated CuFeO_2_ were different from those of a similar reported catalyst which contained metallic Fe and χ-Fe_5_C_2_ without iron oxides^24^. In comparison, the structures of activated Fe_2_O_3_ were also investigated by means of multiple characterization techniques including SEM, XRD, and Mössbauer spectroscopy. We found that activated Fe_2_O_3_ comprised Fe_3_O_4_ and χ-Fe_5_C_2_ (Supplementary Fig. 11 and Supplementary Table 5).

The electronic structures of activated CuFeO_2_ were investigated via X-ray photoelectron spectroscopy (XPS) measurements. Supplementary Figure 12 shows Cu 2*p* XPS spectra of fresh and activated CuFeO_2_. No evident satellite peaks were presented, indicating the absence of Cu^2+^. However, the assignment of Cu^0^ and Cu^+^ cannot be concluded only from XPS results since Cu and Cu^+^ have close binding energies^29,30^. To discriminate between Cu^0^ and Cu^+^, we turned to Cu *LMM* Auger measurements. The Auger spectrum of fresh CuFeO_2_ showed a peak at 917.0 eV which arose from Cu^+^ (Fig. 3d)^29,30^. As for activated CuFeO_2_, two peaks at 916.6 and 918.7 eV were observed, corresponding to Cu^+^ and Cu^0^, respectively (Fig. 3d)^29,30^. Based on Fe 2*p* XPS spectra, Fe species on fresh CuFeO_2_ were at the oxidation state of +3, whereas activated CuFeO_2_ contained Fe^3+^, Fe^2+^, and Fe^0^ species (Fig. 3e)^31,32^. As shown in Fig. 3f, O 1*s* spectra were deconvoluted into three peaks. Specially, the main peaks at around 529.4 and 530.0 eV were ascribed to the lattice O atoms (O_lattice_)^9,33^. The peaks at 531.0 and 531.4 eV were assigned to O atoms proximal to a defect (O_defect_), while those at 532.8 eV corresponded surface hydroxyl groups (OH*)^9,33^.

Based on structural and electronic characterizations, we conclude how CuFeO_2_ reconstructed after the activation procedure. After the activation, Cu^+^ in the lattice of CuFeO_2_ collapsed and aggregated to form pure Cu phase with partially oxidized surface. Meanwhile, Fe^3+^ in the lattice of CuFeO_2_ underwent partial reduction and carbonization, resulting in the formation of Fe_3_O_4_, χ-Fe_5_C_2_, and Fe_3_C. In this case, a mixed states of Fe^3+^, Fe^2+^, and Fe^0^ existed. Therefore, activated CuFeO_2_ was composed of copper, iron oxides, and iron carbides. Moreover, these species were in intimate contact with each other, resulting in the formation of multiple interfacial sites such as the interface between Cu and χ-Fe_5_C_2_.

### Mechanistic studies

**Fig. 4:**
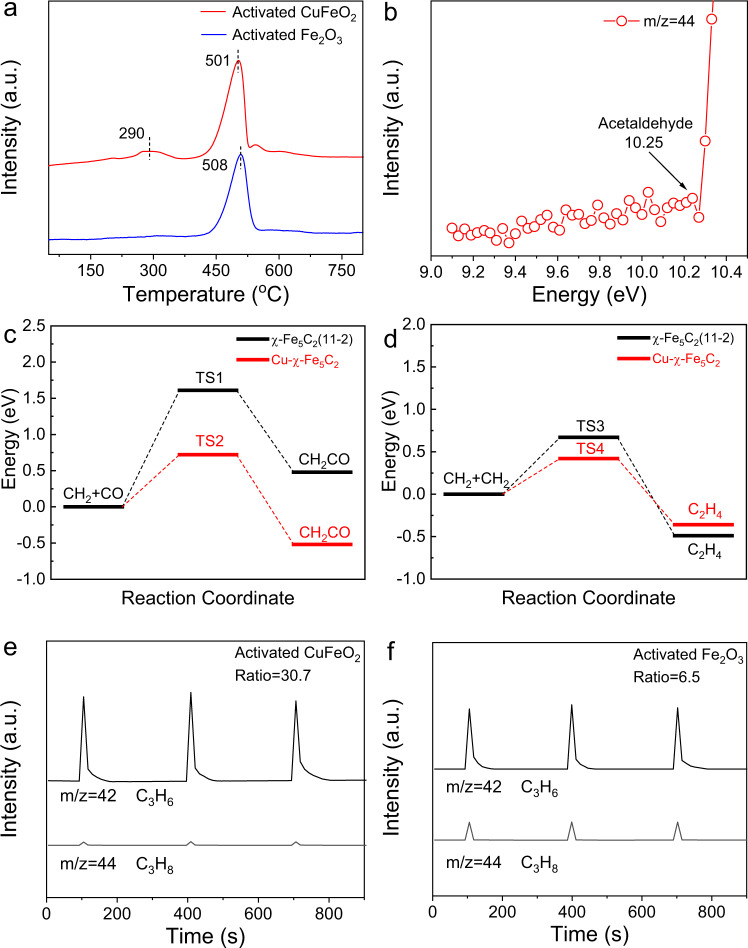
Mechanistic studies. **a** CO-TPD profiles of activated CuFeO_2_ and activated Fe_2_O_3_. **b** Absolute photoionization cross sections for acetaldehyde over activated CuFeO_2_. **c** Comparison in energy barriers of CH_2_ + CO over Cu-χ-Fe_5_C_2_ and χ-Fe_5_C_2_(11-2) facets. **d** Comparison in energy barriers of CH_2_ + CH_2_ over Cu-χ-Fe_5_C_2_ and χ-Fe_5_C_2_(11-2) facets. Transient response curves obtained during propene pulses into H_2_ flow over **e** activated CuFeO_2_ and **f** activated Fe_2_O_3_. Ratio refers to the ratio of C_3_H_6_/C_3_H_8_ peak area detected by mass spectroscopy.

In order to explore the origin of high selectivity for C_4+_^=^ olefins under ambient pressure, we conducted temperature-programmed desorption (TPD) measurements to explore the adsorption of CO. Specially, the samples were exposed to the mixed gas (CO:He = 1:9) under 1 bar at 50 °C for 30 min, followed by being purged in He for 30 min. The TPD curves were recorded from 50 °C to 800 °C at a heating rate of 5 °C min^−1^. The CO-TPD profile of activated Fe_2_O_3_ shows a peak at 508 °C for dissociative adsorption of CO (Fig. 4a)^34^. Considering that activated Fe_2_O_3_ comprised Fe_3_O_4_ and χ-Fe_5_C_2_, we proposed the reaction scheme of activated Fe_2_O_3_ as that for Fe-based catalysts previously reported^16,35,36^. Specially, CO_2_ was hydrogenated over Fe_3_O_4_ into CO via RWGS, while the produced CO was dissociated on χ-Fe_5_C_2_ and subsequently underwent FTS process to yield product whose distribution followed ASF distribution. As for the CO-TPD profile of activated CuFeO_2_, two peaks at 290 and 501 °C appeared, corresponding to non-dissociative and dissociative adsorption of CO, respectively (Fig. 4a)^34,37^. The non-dissociative adsorption of CO* was further supported by diffuse reflectance infrared Fourier transform spectroscopy (DRIFTS) measurements using CO as a probe molecule. Compared with the DRIFTS spectrum of activated Fe_2_O_3_, the DRIFTS spectrum of activated CuFeO_2_ showed an additional peak at 2061 cm^−1^ (Supplementary Fig. 13). This peak was assigned to non-dissociative adsorption of CO*. We further conducted in-situ DRIFTS measurements of activated CuFeO_2_ after exposure to the mixed gas (H_2_:CO_2_ = 3:1, 1 bar) at 300 °C. As shown in Supplementary Fig. 14, the peaks for CH_x_, gaseous CO_2_, gaseous CO, and chemically adsorbed CO* were observed. These spectropic evidences prove non-dissociative adsorption of CO* which serves as a prerequisite for CO insertion.

As activated CuFeO_2_ was composed of Cu, Fe_3_O_4_, χ-Fe_5_C_2_, and Fe_3_C, Fe_3_O_4_ enabled RWGS reaction to produce CO, while CO was non-dissociatively adsorbed on Cu and dissociatively adsorbed on iron carbides, subsequently experiencing FTS process. It is worth noting that the chain propagation process during FTS generally involves CO insertion and carbide mechanism, though all the possible mechanisms can result in the selectivity as predicted by ASF model^38^. Dissociative adsorption of CO favors carbide mechanism which is generally obeyed by C-C coupling over iron carbides^38^. Non-dissociative adsorption of CO allows for CO insertion mechanism, which is implied by the appearance of detectable oxygenated products under high pressure (Supplementary Table 3)^38^.

To identify the existence of CO insertion mechanism, we resorted to probing oxygenate intermediates which are regarded as characteristics of CO insertion, because carbide mechanism cannot account for the formation of oxygenate products via surface polymerization of CH_x_^39^. To detect the intermediate, we conducted synchrotron-based vacuum ultraviolet photoionization mass spectrometry (SVUV-PIMS). This technique is highly sensitive to low-concentration intermediates and able to identify isomers because of tunable photon energy and soft ionization^40,41^. The reaction proceeded under 1 bar (H_2_:CO_2_ = 3:1) with a high space velocity of 36,000 mL h^−1^ g_cat_^−1^ at 320 °C to shorten the contact time in case that active intermediates would be hydrogenated. The photon energy was selected at 10.40 eV to avoid the ionization of CO_2_ whose ionization energy is 13.77 eV^42^. For activated CuFeO_2_, a signal of mass/charge ratio (m/z) = 44 appeared in addition to stable hydrocarbon products (Supplementary Fig. 15). When the photon energy was varied from 9.10 to 10.40 eV, the point of inflection appeared at 10.25 eV which was assigned to acetaldehyde (CH_3_CHO) after excluding the possibilities of other species with m/z = 44 (Fig. 4b and Supplementary Table 6)^43^. To investigate whether C-C coupling occurs via acetaldehyde oligomerization, we conducted temperature-programmed surface reaction (TPSR) measurements of activated CuFeO_2_. Specially, the acetaldehyde vapor was introduced via bubbling with the mixed gas (H_2_:Ar = 1:9, 1 bar) with a gas-flow rate of 100 mL min^−1^. Then the temperature was raised to 300 °C with a rate of 10 °C min^−1^ and kept at 300 °C. During the whole period, no detectable products were observed as shown in Supplementary Fig. 16, thereby excluding the possibility of the acetaldehyde oligomerization. Therefore, we speculate that CO insertion occurred during the chain growth over activated CuFeO_2_.

Generally, interfacial sites between copper and iron carbides are regarded as the active center for CO insertion. To gain theoretical insights into the mechanisms, we conducted density functional theory (DFT) calculations. To simulate the interface between iron carbides and copper, we established χ-Fe_5_C_2_ clusters on the surface of Cu(111) (Supplementary Fig. 17). This simplified model captures the main features of the interface, though this model cannot completely reproduce the real catalyst. Cu(111) and χ-Fe_5_C_2_(11-2) were constructed for comparison (Supplementary Fig. 17). We calculated the *d*-band centers of these facets to qualitatively evaluate the catalytic performance. The *d*-band center of Cu-χ-Fe_5_C_2_ interfacial sites is −0.32 eV, which is closer to the Fermi level than that (−0.69 eV) of χ-Fe_5_C_2_(11-2) and that (−2.02 eV) of Cu(111) facets (Supplementary Fig. 18). The upward shift of *d*-band center empties more antibonding states which allows for accepting more electrons from CO and alkyl species, thereby facilitating CO insertion into alkyl species^44,45^.

To further investigate CO insertion over different surfaces, we calculated the energy barriers of C–C coupling. FTS is a complex process involving various possible steps. For simplicity, we use CH_2_ + CO to represent the C-C coupling via CO insertion, while CH_2_ + CH_2_ represents the carbide mechanism. CO insertion over χ-Fe_5_C_2_(11-2) is an endothermic process (Δ*H* = + 0.48 eV), requiring an energy barrier as high as 1.61 eV (Fig. 4c and Supplementary Fig. 19). As for Cu-χ-Fe_5_C_2_ interfacial sites, CO insertion becomes exothermic (Δ*H* = −0.52 eV), while the energy barrier decreases to 0.72 eV (Fig. 4c and Supplementary Fig. 20). As such, Cu-χ-Fe_5_C_2_ interface significantly promotes CO insertion relative to iron alone. In contrast, the carbide mechanism is less sensitive to interface than CO insertion, as the introduction of interface only lowers the energy barrier by 0.25 eV (*versus* 0.89 eV in CO insertion) (Fig. 4c, d, Supplementary Figs. 19–22).

To support the important role played by interface, we qualitatively tuned the interfacial sites by varying the ratios of Cu to Fe from 1:9 to 9:1 in Cu-Fe binary oxides. Though we cannot quantify the interfacial sites, the amount of interfacial sites should exhibit a volcano-type trend maximized at Cu:Fe = 1:1 based on rough estimation. The reaction was conducted under 1 bar (H_2_:CO_2_ = 3:1) with a space velocity of 2,400 mL h^−1^ g_cat_^−1^ at 320 °C after 4 h on stream. As shown in Supplementary Figure 23, the selectivity for C_4+_^=^ exhibited a volcano-type trend against the Cu:Fe ratios with the maximum at Cu:Fe = 1:1 (activated CuFeO_2_). We can draw a qualitative conclusion that the C_4+_^=^ selectivity increased with Cu-Fe interfacial sites. This result indirectly supported our claim that interfacial sites promoted C-C coupling. Therefore, the synergy of carbide path over iron carbides and CO insertion path over copper/iron carbides interfacial sites led to efficient C-C coupling into long-chain products under ambient pressure (Supplementary Fig. 24).

Given that Cu–Fe interfacial sites contribute to C–C coupling, we explain the deactivation and regeneration mechanisms. The overlap between Cu and Fe elements can roughly estimate the amount of interfacial sites. The results show that the 20-h reaction under ambient pressure led to a significant decrease in the interfacial sites between copper and iron. Moreover, the regeneration treatment redispersed Cu and Fe elements, leading to the recovery of interfacial sites. As such, the decreased C_4+_ selectivity with the reaction time presumably derived from the decreased interfacial sites. The regeneration not only cleaned the carbon deposits but also recovered the interfacial sites, inducing the recovery of the C_4+_ selectivity.

To explain the high o/p ratio of activated CuFeO_2_, we turned to pulse experiments for exploring whether alkenes prefer desorption or hydrogenation on the catalysts. The catalysts were operated under 1 bar (H_2_:CO_2_ = 3:1) at 320 °C for 1 h, before switching to a H_2_ flow. Afterward, propene which was chosen as the representative of alkenes was pulsed into the reactor. As shown in Fig. 4e, f, the ratio of C_3_H_6_/C_3_H_8_ peak area for activated CuFeO_2_ was 30.7, much higher than that (6.5) for activated Fe_2_O_3_. Such a high ratio for activated CuFeO_2_ indicates that the formation of propane was almost totally inhibited.

In conclusion, we achieved ambient-pressure hydrogenation of CO_2_ into long-chain olefins over activated CuFeO_2_. The C_4+_^=^ selectivity under 1 bar at 320 °C reached as high as 66.9% which was comparable to that of the state-of-the-art catalysts optimized under high pressure (35 bar). The high C_4+_^=^ selectivity under ambient pressure derives from the synergy of carbide path and CO insertion path. Our findings represent a promising approach to CO_2_ conversion connected with a decentralized use of renewable power-generated hydrogen. Moreover, we offer a viable method for the regeneration of deactivated catalysts under ambient-pressure hydrogenation of CO_2_.

## Methods

### Chemicals and materials

Fe(NO_3_)_3_·9H_2_O, Cu(NO_3_)_2_·3H_2_O, NaOH, Na_2_CO_3_, (NH_4_)_2_CO_3_, Zn(NO_3_)_2_·6H_2_O, Zr(NO_3_)_2_·5H_2_O and FeCl_3_ were analytical grade and purchased from Sinopharm Chemical Reagent Co., Ltd. FeCl_2_·4H_2_O and acetaldehyde aqueous solution (35%) were purchased from Sigma-Aldrich. SAPO-34 zeolites was purchased from Nankai University Catalyst Co., Ltd.

### Synthesis of CuFeO_2_

CuFeO_2_ was synthesized via a hydrothermal method. Typically, 2.42 g of Cu(NO_3_)_2_·3H_2_O and 4.04 g of Fe(NO_3_)_3_·9H_2_O were added to 40 mL of deionized (DI) water with stirring to form a clear solution. In the above solution, 40 mL of 5 M NaOH aqueous solution was then added dropwise under stirring at room temperature. After stirring for 30 min, 1 mL of propionaldehyde was added as the reducing agent. The mixture was transferred to 100-mL Teflon-lined stainless steel autoclave and kept at 180 °C for 24 h. The product was separated by centrifugation, washed twice with DI water, and dried overnight at 60 °C.

### Synthesis of activated CuFeO_2_

Five hundred milligrams of CuFeO_2_ was reduced in 4 bar of a pure H_2_ flow with a flow rate of 100 mL min^−1^ at 400 °C for 2 h, followed by being exposed to 1 bar of mixed gas (H_2_:CO_2_ = 3:1) with a space velocity of 2,400 mL h^−1^ g_cat_^−1^ at 320 °C for 4 h.

### Synthesis of Fe_2_O_3_

Fe_2_O_3_ was synthesized via co-precipitation method. Typically, 8.08 g of Fe(NO_3_)_3_·9H_2_O was added to 40 mL of DI water under stirring until the formation of a clear solution. In the above solution, 20 mL of 2 M Na_2_CO_3_ aqueous solution was then added dropwise under stirring at room temperature. After being aged for 1 h, the turbid liquid was filtrated, washed twice with DI water, and dried overnight at 60 °C. The resulting powders were calcinated in muffle furnace at 350 °C for 4 h.

### Synthesis of activated Fe_2_O_3_

Five hundred milligrams of Fe_2_O_3_ was reduced in 4 bar of a pure H_2_ flow with a flow rate of 100 mL min^−1^ at 400 °C for 2 h, followed by being exposed to 1 bar of mixed gas (H_2_:CO_2_ = 3:1) with a space velocity of 2,400 mL h^−1^ g_cat_^−1^ at 320 °C for 4 h.

### Synthesis of ZnZrO/SAPO

ZnZrO was synthesized via co-precipitation method, Typically, 1.325 g of Zn(NO_3_)_2_·6H_2_O and 5.66 g of Zr(NO_3_)_4_·5H_2_O were dissolved in 70 mL of DI water at 70 °C. 50 mL of 0.625 M (NH_4_)_2_CO_3_ aqueous solution was added dropwise under vigorous stirring at 70 °C. The pH value of the solution was kept at about 7.0. After being aged for 2 h at 70 °C, the product was separated by centrifugation, washed twice with DI water, and dried overnight at 80 °C. The resulting powders were calcinated in muffle furnace at 500 °C for 5 h. The ZnZrO/SAPO was prepared through physical mixing the ZnZrO solid solution and SAPO-34 zeolite, the mass ratio of these two components was 1:1.

### Synthesis of Cu-Fe binary oxide with different ratios

copper iron binary oxide was synthesized via co-precipitation method, 2.42 g Cu(NO_3_)_2_·3H_2_O and Fe(NO_3_)_3_·9H_2_O with a given molar ratio of Cu^2+^:Fe^3+^ (9:1, 3:1, 1:3, 1:9, respectively) were dissolved in 50 mL of DI water with stirring to form a clear solution. In the above solution, 2 M Na_2_CO_3_ aqueous solution was then added dropwise under stirring at room temperature, and the pH value of final suspension was maintained at 9. After being aged for 1 h, the product was separated by centrifugation, washed twice with DI water, and dried overnight at 60 °C. The resulting powders were calcinated in muffle furnace at 350 °C for 4 h.

### Catalytic tests

CO_2_ hydrogenation reactions were carried out in a fixed-bed reactor under 1 bar of mixed gas at 320 °C. The mixed gas contained 96 vol% H_2_/CO_2_ as reactants and 4 vol% Ar as an internal standard. Generally, the catalyst (500 mg, 20–40 meshes) diluted with powdered quartz (500 mg, 20–40 meshes) was loaded into a fixed-bed reactor with an inner diameter of 9 mm. The catalysts refer to activated CuFeO_2_ and activated Fe_2_O_3_.

For the tests over ZnZrO/SAPO, 200 mg of ZnZrO/SAPO was pretreated in an Ar flow with a flow rate of 30 mL min^−1^ at 380 °C for 1 h. Afterward, the catalyst was exposed to 1 bar of mixed gas (H_2_:CO_2_ = 3:1) with a space velocity of 2,400 mL h^−1^ g_cat_^−1^ at 380 °C. The catalytic data were obtained after when the reaction reached a steady state.

For the tests over Cu-Fe binary oxides with different ratios, 500 mg of the catalyst (20–40 meshes) was reduced in 4 bar of a pure H_2_ flow with a flow rate of 100 mL min^−1^ at 400 °C for 2 h. followed by being exposed to 1 bar of mixed gas (H_2_:CO_2_ = 3:1) with a space velocity of 2,400 mL h^−1^ g_cat_^−1^ at 320 °C for 4 h.

All of the products from the reactor were introduced in a gaseous state and analyzed with two online gas chromatographs (Shimadzu GC-2014). H_2_, CO, CO_2_, CH_4_, and Ar were analyzed by using a carbon molecular sieves column (TDX-1) with a thermal conductivity detector (TCD). Hydrocarbons were analyzed using a PONA capillary column with a flame ionization detector (FID). CH_4_ was taken as a reference bridge between TCD and FID. CO_2_ conversion was calculated according to an internal standard method, assuming that the amount of Ar remained constant after the reaction.

CO_2_ conversion was calculated on a carbon-atom basis, as follows:

1 $${{{{{{\rm{CO}}}}}}}_{2}\,{{{{{\rm{conversion}}}}}}=\frac{{{{{{\rm{CO}}}}}}_{{{{{\rm{2inlet}}}}}}-{{{{{\rm{CO}}}}}}_{{{{{\rm{2outlet}}}}}}}{{{{{{\rm{CO}}}}}}_{{{{{\rm{2inlet}}}}}}}\times 100 \%$$

where CO_2 inlet_ and CO_2 outlet_ are moles of CO_2_ at the inlet and outlet, respectively.

CO selectivity was calculated according to:

2 $${{{{{\rm{CO}}}}}}\,{{{{{\rm{selectivity}}}}}}=\frac{{{{{{\rm{CO}}}}}}_{{{{{\rm{outlet}}}}}}}{{{{{{\rm{CO}}}}}}_{{{{{\rm{2inlet}}}}}}-{{{{{\rm{CO}}}}}}_{{{{{\rm{2outlet}}}}}}}\times 100 \%$$

where CO_outlet_ refers to moles of CO at the outlet.

The selectivity for hydrocarbon C_n_H_m_ was obtained according to:

3 $${{{{{{\rm{C}}}}}}}_{{{{{{\rm{n}}}}}}}{{{{{{\rm{H}}}}}}}_{{{{{{\rm{m}}}}}}}\,{{{{{\rm{selectivity}}}}}}=\frac{{n}{{{{{{{\rm{C}}}}}}}_{{{{{{\rm{n}}}}}}}{{{{{{\rm{H}}}}}}}_{{{{{{\rm{m}}}}}}\,{{{{{\rm{outlet}}}}}}}}}{\mathop{\sum}\limits_{i}i{{{{{{\rm{C}}}}}}}_{{{{{{\rm{i}}}}}}}{{{{{{\rm{H}}}}}}}_{{{{{{\rm{m}}}}}}\,{{{{{\rm{outlet}}}}}}}}\times 100 \%$$

where C_n_H_m outlet_ represents moles of individual hydrocarbon product at the outlet. The selectivity for oxygenates was below 1.0% and therefore was not reported in the product selectivity. The carbon balance was over 95.0%.

### Mössbauer measurements

^57^Fe Mössbauer spectra were carried out on a Topologic 500 A spectrometer driving with a proportional counter at room temperature. The radioactive source was ^57^Co (Rh) moving in a constant acceleration mode. Data analyses were performed assuming a Lorentzian lineshape for computer folding and fitting.

### TGA

TGA was conducted on Pyris Diamond TG-DTG in a N_2_ flow with the rate of 100 mL min^−1^ at the heating rate of 5 °C min^−1^ from 50 °C to 800 °C.

### CO-TPD measurements

CO-TPD measurements were conducted by using a TPD instrument (AutoChem II 2920). Prior to CO-TPD, the samples were cleaned in He with a flow rate of 50 mL min^−1^ at 200 °C for 2 h. Then, the gas was switched to the mixed gas (CO:He = 1:9, 1 bar)with a flow rate of 20 mL min^−1^ at 50 °C. After CO adsorption for 30 min, the samples were purged by He with a flow rate of 50 mL min^−1^ at 50 °C for 30 min. The CO-TPD curves were recorded from 50 °C to 800 °C at a heating rate of 5 °C min^−1^.

### DRIFTS spectra using CO as a probe molecule

In-situ DRIFTS experiments were conducted in an elevated-pressure cell (DiffusIR Accessory PN 041-10XX) with a Fourier transform infrared spectrometer (TENSOR II Sample Compartment) and a liquid-nitrogen-cooled MCT detector. Spectra were measured by accumulating 32 scans at a resolution of 8 cm^−1^. Prior to the test, the sample was flushed with He with a gas-flow rate of 30 mL min^−1^ at 200 °C for 30 min, followed by cooling to 25 °C. The background spectra of the sample were acquired under He flow at 25 °C. Then, 1 bar (CO:He = 1:9) with a gas-flow rate of 10 mL min^−1^ was allowed to flow into the cell at 25 °C for 30 min. The spectra were recorded under the mixed gas (CO:He = 1:9).

### In-situ DRIFTS spectra of activated CuFeO_2_

In-situ DRIFTS experiments were conducted in an elevated-pressure cell (DiffusIR Accessory PN 041-10XX) with a Fourier transform infrared spectrometer (TENSOR II Sample Compartment) and a liquid-nitrogen-cooled MCT detector. Spectra were measured by accumulating 32 scans at a resolution of 8 cm^−1^. Prior to the test, the sample was reduced in 1 bar of H_2_ with a gas-flow rate of 50 mL min^−1^ at 300 °C for 30 min. Afterward, the sample was flushed with He with a gas-flow rate of 30 mL min^−1^ at 300 °C for 30 min. The background spectra of the sample were acquired under He flow at 300 °C. Then, 1 bar (H_2_:CO_2_ = 3:1) with a gas-flow rate of 20 mL min^−1^ was allowed to flow into the cell at 300 °C for 30 min, followed by purged with 1 bar of He with a gas-flow rate of 30 mL min^−1^ at 300 °C and the spectra were obtained to detect the adsorbed species on the sample.

### SVUV-PIMS

SVUV-PIMS study was carried out at the combustion beamline of the National Synchrotron Radiation Laboratory at Hefei, China. A quartz reactor with a nozzle size of ~0.1 mm was designed, which was connected to the online SVUV-PIMS spectrometer. The detection limit of SVUV-PIMS is 0.1 ppm for CO_2_. The reaction proceeded under 1 bar (H_2_:CO_2_ = 3:1) with a space velocity of 36,000 mL h^−1^ g_cat_^−1^ at 320 °C.

### TPSR measurement of activated CuFeO_2_

TPSR measurement was carried out in a AutoChem II 2920 apparatus with a mass spectrometer (Hiden HPR20). The activated CuFeO_2_ (500 mg, 20–40 meshes) was loaded in a quartz U-tube. After pretreatment with He at 200 °C for 30 min, the acetaldehyde vapor was introduced by bubbling with 1 bar (H_2_:Ar = 1:9) with a gas-flow rate of 100 mL min^−1^. Then the temperature was raised to 300 °C with a rate of 10 °C min^−1^ and kept at 300 °C.

### DFT methods

DFT calculations were performed using the Vienna ab initio Simulation Package code^46–48^. The Perdew-Burke-Ernzerhof functional with generalized gradient approximation^49^ was used for the geometry optimizations and electronic structure calculations. The projector-augmented wave method^50^ was used to describe the electron-ion interactions. The atomic structures were fully relaxed by using a conjugate gradient scheme without symmetry restrictions until the maximum force on each atom was less than 0.02 eV Å^−1^. A vacuum space of ~20 Å along the *z* direction was used to separate the interactions between the neighboring slabs with an energy cutoff of 500 eV. The Cu(111) surface containing 96 Cu atoms is simulated by using a (4 × 6) supercell (10.224 Å × 13.281 Å) with four Cu layers, where the lower two layers are fixed to the bulk structure, while the upper two layers are fully relaxed. The DFT calculated lattice constants of bulk χ-Fe_5_C_2_ are (a = 11.354 Å, *b* = 4.413 Å, *c* = 4.914 Å; *α* = γ = 90^o^, *β* = 97.61^o^). The χ-Fe_5_C_2_(11-2) surface is simulated using a (1 × 1) supercell which contains 80 Fe and 32 C atoms. The Cu-χ-Fe_5_C_2_ interface is constructed from a (4 × 6) Cu(111) surface and a cluster of Fe_10_C_4_. The (3 × 3 × 1) Monkhorst-Pack mesh is used to sample the Brillouin-zone for all the electronic structure calculations. The (1 × 1 × 1) *k*-mesh is used for the geometry optimizations.

### TPD pulse experiments

TPD pulse experiments were carried out in a AutoChem II 2920 apparatus with a mass spectrometer (Hiden HPR20). 200 mg of activated catalysts were cleaned in He with a gas-flow rate of 50 mL min^−1^ at 200 °C for 2 h, followed by treated in 1 bar of mixed gas (H_2_:CO_2_ = 3:1) with a flow rate of 20 mL min^−1^ at 320 °C. After 1-h reaction, the gas was switched to the mixed gas (H_2_:He = 1:9) with a flow rate of 50 mL min^−1^. 525 μL of diluted (C_3_H_6_:He = 1:9) was pulsed into the system every 5 min.

### Characterizations

XRD patterns were recorded by using a Philips X’Pert Pro Super diffractometer with Cu-Kα radiation (λ = 1.54178 Å). XPS measurements were conducted on an ESCALAB 250 (Thermo-VG Scientific, USA) with an Al Kα X-ray source (1486.6 eV protons) in Constant Analyser Energy (CAE) mode with pass energy of 30 eV for all spectra. The values of binding energies were calibrated with the C1s peak of contaminant carbon at 284.60 eV. Raman spectra were detected by a Renishaw. RM3000 Micro-Raman system with a 514.5 nm Ar laser.

## Supplementary information

Supplementary Information

## Data Availability

The data generated in this study are provided in the Supplementary Information/Source Data file. Source data are provided with this paper.

## Source data

Source Data
