# Supplementary material for: Ambient-pressure hydrogenation of CO2 into long-chain olefins
Source: Nat Commun. 2022 May 3;13:2396. doi: 10.1038/s41467-022-29971-5 (PMC9064975; doi:10.1038/s41467-022-29971-5)
Supplement: Supplementary file 1 — Supplementary Information [file 41467_2022_29971_MOESM1_ESM.pdf]

Supplementary Information for

Ambient-pressure hydrogenation of CO_2 into long-chain olefins

Zhongling Li[1†], Wenlong Wu[1†], Menglin Wang[1], Yanan Wang[2,3], Xinlong Ma[1], Lei Luo[1], Yue Chen[1], Kaiyuan Fan[1], Yang Pan[1], Hongliang Li[1*], Jie Zeng[1*]

[1]Hefei National Laboratory for Physical Sciences at the Microscale, CAS Key Laboratory of Strongly-Coupled Quantum Matter Physics, National Synchrotron Radiation Laboratory, Key Laboratory of Surface and Interface Chemistry and Energy Catalysis of Anhui Higher Education Institutes, Department of Chemical Physics, University of Science and Technology of China, Hefei, Anhui 230026, P. R. China

[2]Songshan Lake Materials Laboratory, Dongguan, Guangdong 523808, P. R. China

[3]Beijing National Laboratory for Condensed Matter Physics and Institute of Physics, Chinese Academy of Sciences, Beijing 100190, P. R. China

[†]These authors contributed equally: Zhongling Li, Wenlong Wu.

[*]e-mail: lihl@ustc.edu.cn; zengj@ustc.edu.cn.

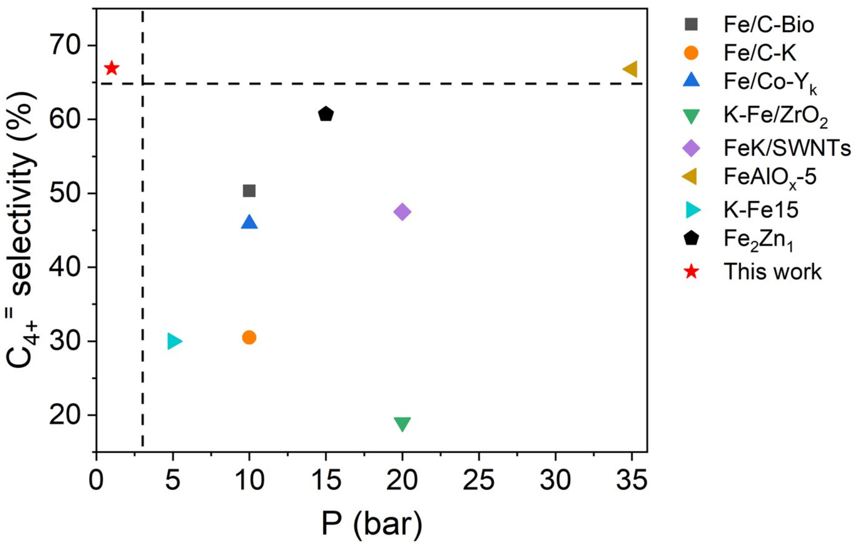

Supplementary Figure 1 | Comparison of $C_{4+}^{=}$ selectivity. Detailed information is listed in Supplementary Table 1. Horizontal dash line represents $C_{4+}^{=}$ selectivity of 65.0%, while vertical dash line represents total pressure of 3 bar.

Supplementary Table 1 | Comparison of catalytic properties.

Catalyst	P (bar)	H_2:CO_2 (v/v)	T (°C)	CO_2 conv. (%)	CO sel. (%)	Hydrocarbon distribution (%)				
						CH_4	C_2-$C_3^=$	C_2-C_3^o	$C_{4+}^=$	C_{4+}^o
Fe/C-Bio[1]	10	3	320	31.0	23.2	11.8	21.7	2.7	50.3	13.5
Fe/C-K[1]	10	3	320	28.0	22.6	24	29.9	8.2	30.5	7.4
Fe/Co-Y_k[2]	10	3	300	25.9	21.1	13.9	25.0	5.2	45.9	10
K-Fe/ZrO_2[3]	20	3	340	43.0	15.0	18	44.0[a]	9.2[b]	19.0[c]	9.8[d]
FeK/SWNTs[4]	20	3	340	52.7	9.6	13.5	10.0	-	47.5	-
$FeAlO_x$-5[5]	35	1	330	20.2	16.8	5.4	11.7	-	66.8	-
K-Fe15[6]	5	3	300	45.0	12.5	18.3	42.3	-	30.0	-
Fe_2Zn_1[7]	15	3	330	43.5	9.2	11.5	-	-	60.7	7.5
ZnCrO/SAPO[8]	20	3	380	12.6	47	3	80[a]	14[b]	3[e]	
ZnCrO/SAPO (This work)	1	3	380	3.8	85.1	30.3	61.4[a]	8.3[b]	0[c]	0[d]
							57.9	7.8	3.5	0.5
Activated Fe_2O_3 (This work)	30[e]	3	320	30.8	11.2	7.0	20.7	2.9	60.2	9.2
	1[e]	3	320	26.6	84.5	17.9	31.8	4.3	40.4	5.6
Activated $CuFeO_2$ (This work)	1[e]	3	320	27.3	43.7	5.4	18.8	1.8	66.9	7.1
	1[f]	3	320	25.8	46.5	5.7	21.4	1.9	65.1	5.9

[a]C_2–$C_4^=$ products. [b]C_2–C_4^0 products. [c]$C_{5+}^=$ products. [d]C_{5+}^0 products. [e]Space velocity = 2,400 mL h^{-1} g_{cat}^{-1}. [f]Space velocity = 4,800 mL h^{-1} g_{cat}^{-1}.

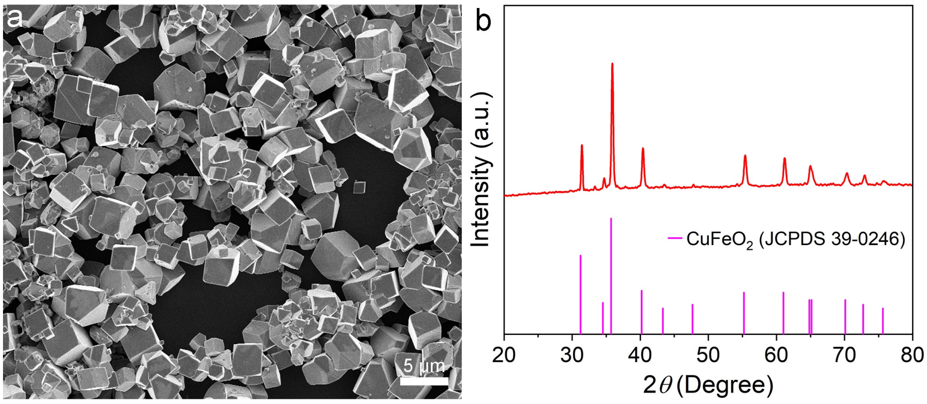

Supplementary Figure 2 | Structural characterizations of $CuFeO_2$. (**a**) SEM image of fresh $CuFeO_2$. (**b**) XRD profile of fresh $CuFeO_2$.

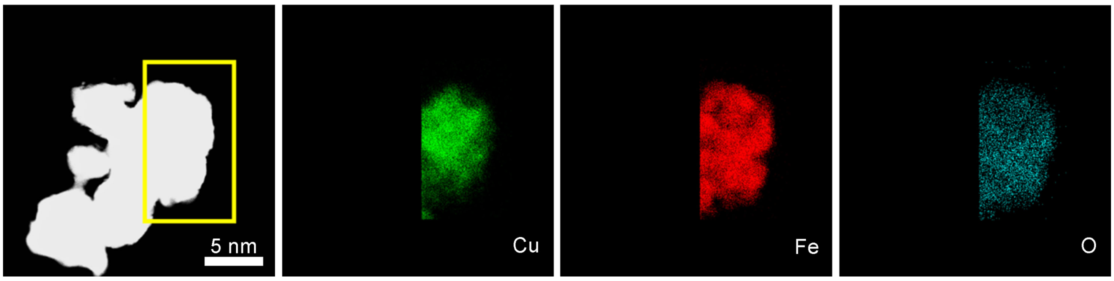

Supplementary Figure 3 | Elemental mapping analysis of fresh $CuFeO_2$. STEM image and STEM-EDX elemental mapping of Cu, Fe, and O elements of fresh $CuFeO_2$.

Supplementary Table 2 | Catalytic properties of activated $CuFeO_2$ under high pressures.

Catalyst	P (bar)	T (°C)	GHSV (mL g^{-1} h^{-1})	CO_2 conv. (%)	CO sel. (%)	Oxy-Sel. (%)	Hydrocarbon distribution (%)			o/p
							CH_4	C_2-C_4	C_{5+}	
$CuFeO_2$-6[9]	10	300	1,800	17.3	31.7	-	2.7	31	66.3[a]	7.3[d]
Activated $CuFeO_2$ (This work)	10	300	1,800	21.4	32.4	3.2	7.5	31.3	61.2[b]	7.7[e]
	30	300	1,800	27.5	14.1	7.1	6.2	29.4	64.4[c]	6.1[e]

[a]The C_{5+} products cover the gasoline (C_5-C_{11}), diesel (C_{12}-C_{21}) ranges, and ~15% of waxy hydrocarbons (C_{25+}), The main product is C_{12+} hydrocarbons.

[b]The data in our work. The C_{5+} products cover 55.7% of C_5-C_{11}, and 5.5% of C_{12+} hydrocarbons.

[c]The data in our work. The C_{5+} products cover 57.3% of C_5-C_{11}, and 7.1% of C_{12+} hydrocarbons.

[d]Olefins/paraffin ratio (o/p) of C_2-C_4 products.

[e]Olefins/paraffin ratio (o/p) of C_{2+} products.

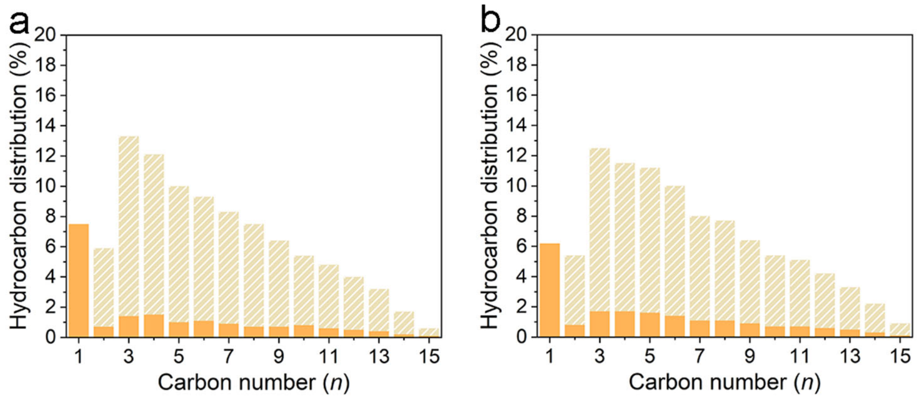

Supplementary Figure 4 | Comparison in the detailed hydrocarbon product distributions. (**a**, **b**) hydrocarbon product distribution of activated $CuFeO_2$ under 10 bar and 30 bar. H_2:CO_2 = 3:1; Space velocity = 1,800 mL h^{-1} g_{cat}^{-1}; T = 300 °C.

Supplementary Table 3. Catalytic properties of activated $CuFeO_2$. Space velocity = 2,400 mL h^{-1} g_{cat}^{-1}. T = 320 °C.

Catalyst	P (bar)	CO_2 conv. (%)	CO sel. (%)	Oxy-sel. (%)	Hydrocarbon distribution (%)				
					CH_4	C_2-$C_3^{=}$	C_2-C_3^{o}	$C_{4+}^{=}$	C_{4+}^{o}
Activated $CuFeO_2$[a]	30	31.7	12.3	7.5	5.8	14.3	2.3	66.3	11.3
Activated $CuFeO_2$[a]	1	27.3	43.7	-	5.4	18.8	1.8	66.9	7.1
Activated $CuFeO_2$[b]	1	26.8	46.3	-	5.7	19.3	1.9	66.5	6.6

[a]The catalyst was pre-reduced in 4 bar of H_2.

[b]The catalyst was pre-reduced in 1 bar of H_2.

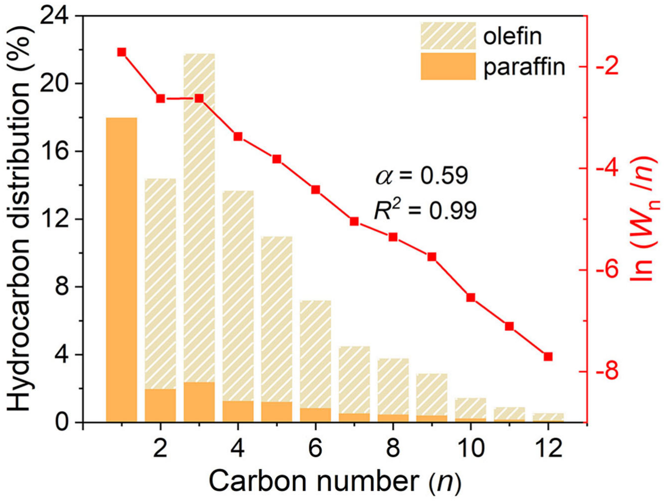

Supplementary Figure 5 | The detailed hydrocarbon product distribution, the ASF plot, and the corresponding α value of activated Fe_2O_3. α is the probability of chain growth. R^2 is the coefficient of determination, describing the goodness of linear fitting. W_n is the weight fraction of a product with n carbon atoms. The reaction was conducted under 1 bar (H_2:CO_2 = 3:1) with a space velocity of 2,400 mL h^{-1} g_{cat}^{-1} at 320 °C. Time on stream = 4 h.

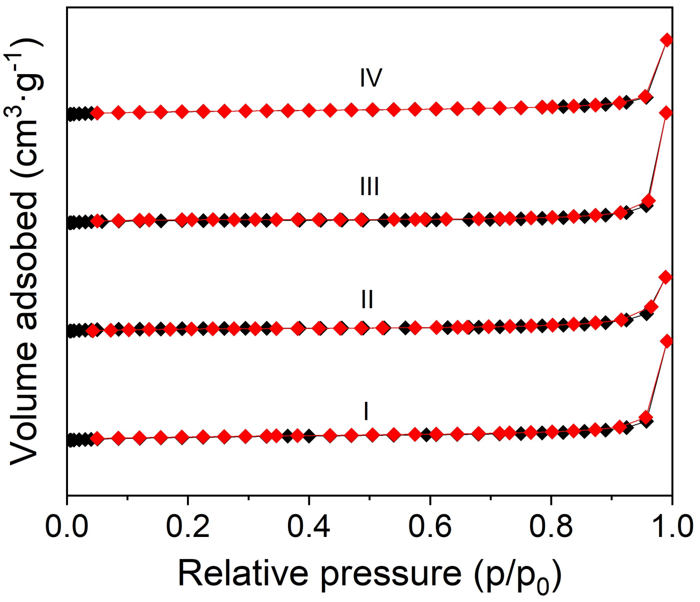

Supplementary Figure 6 | N_2 adsorption-desorption isotherm of activated $CuFeO_2$ with different reaction times. Samples I, II, III, and IV represent the activated $CuFeO_2$ after reaction for 0 h, 10 h, 20 h, and regeneration, respectively. The BET surface areas of samples I, II, III, and IV were measured as 2.88 m^2 g^{-1}, 2.62 m^2 g^{-1}, 2.35 m^2 g^{-1}, and 2.96 m^2 g^{-1}, respectively.

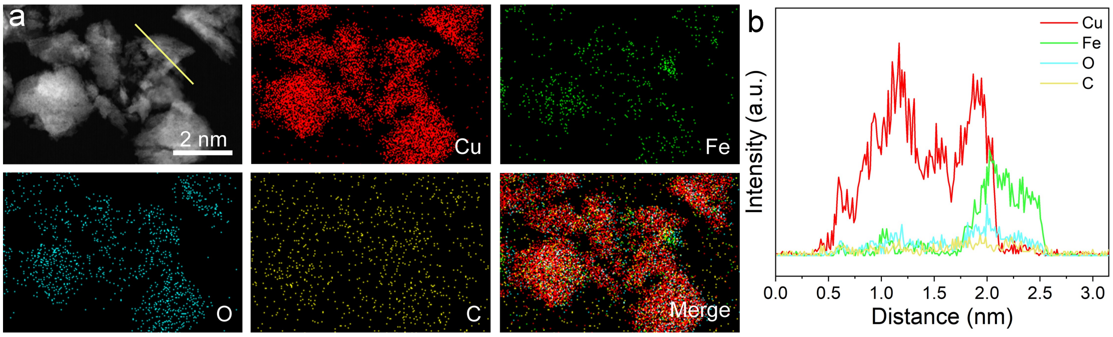

Supplementary Figure 7 | Compositional characterizations of activated $CuFeO_2$. (**a**) HAADF-STEM image and STEM-EDX elemental mapping images of activated $CuFeO_2$. (**b**) Compositional line profile recorded along the line shown in the panel **a**.

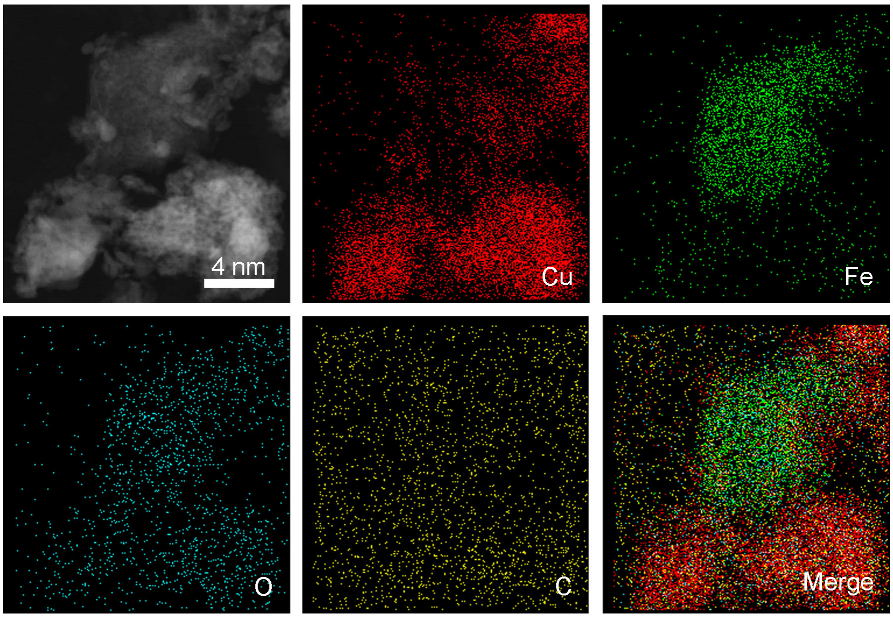

Supplementary Figure 8 | Compositional characterizations of activated $CuFeO_2$ after reaction for 20 h. 1 bar; H_2:CO_2 = 3:1; Space velocity = 2,400 mL h^{-1} g_{cat}^{-1}; 320 °C.

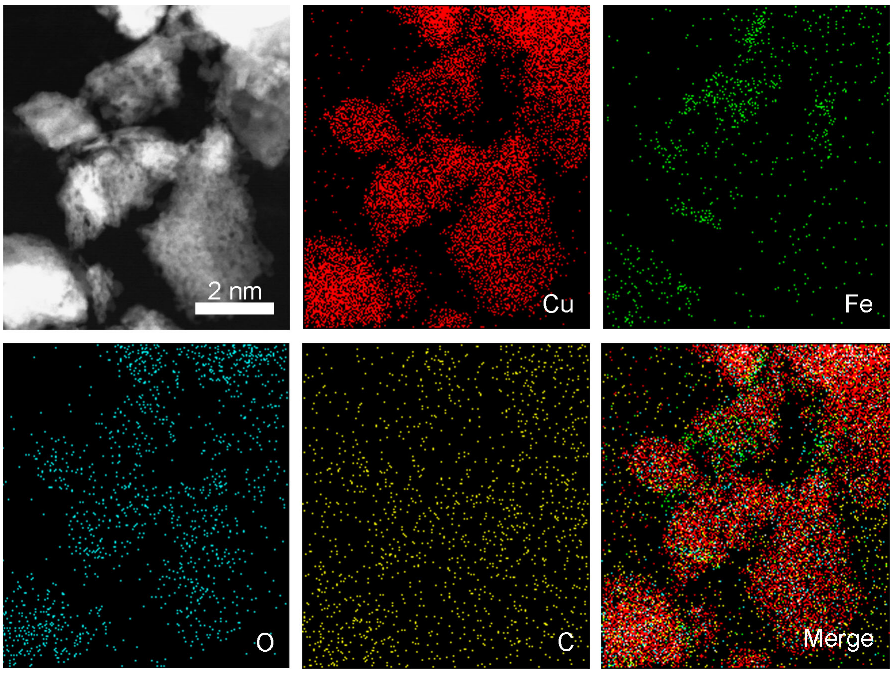

Supplementary Figure 9 | Compositional characterizations of activated $CuFeO_2$ after regeneration treatment. Regeneration treatment was conducted under 30 bar (H_2:CO_2 = 3:1) with a space velocity of 2,400 mL h^{-1} g_{cat}^{-1} at 320 °C.

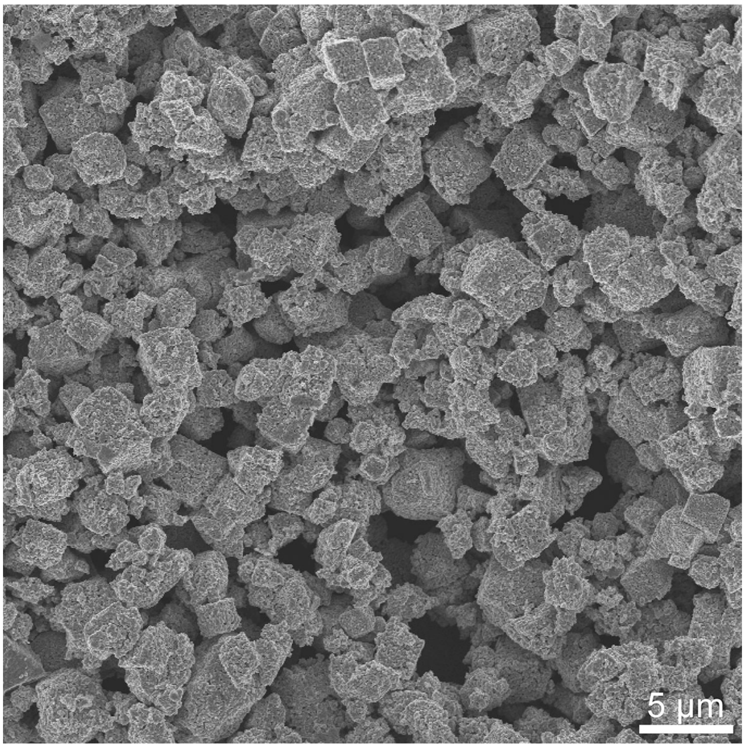

Supplementary Figure 10 | SEM image of activated $CuFeO_2$.

Supplementary Table 4 | Detailed Mössbauer parameters. IS represents isomer shift, while QS represents quadrupole splitting.

Figure	Assignment	Mössbauer parameters			
		IS (mm/s)	QS (mm/s)	Magnetic field (T)	Spectral Contribution (%)
Fig. 3c	Fe_3O_4 (A)	0.28	0.01	49.0	5.9
	Fe_3O_4 (B)	0.67	0.01	45.9	10.7
	χ-Fe_5C_2 (I)	0.24	0.1	21.6	30.9
	χ-Fe_5C_2 (II)	0.16	0.07	18.4	26.8
	χ-Fe_5C_2 (III)	0.23	0.11	11.0	15.9
	Fe_3C	0.18	0.02	20.0	9.8

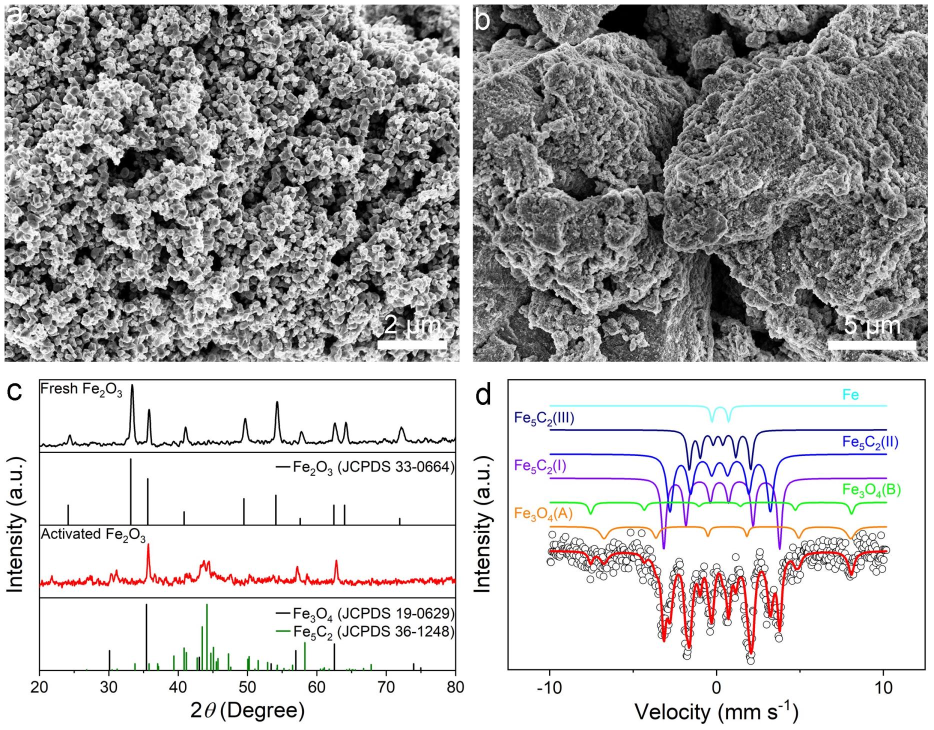

Supplementary Figure 11 | Structural characterizations of Fe_2O_3. (**a**) SEM image of fresh Fe_2O_3. (**b**) SEM image of activated Fe_2O_3. (**c**) XRD profiles of fresh and activated Fe_2O_3. (**d**) Mössbauer spectra of activated Fe_2O_3.

Supplementary Table 5 | Detailed Mössbauer parameters. IS represents isomer shift, while QS represents quadrupole splitting.

Figure	Assignment	Mössbauer parameters			
		IS (mm/s)	QS (mm/s)	Magnetic field (T)	Spectral Contribution (%)
Supplementary Fig. 6d	Fe_3O_4 (A)	0.24	0.08	48.6	5.0
	Fe_3O_4 (B)	0.65	0.01	45.9	9.6
	χ-Fe_5C_2 (I)	0.25	0.13	21.6	31.4
	χ-Fe_5C_2 (II)	0.20	0.03	18.7	35.5
	χ-Fe_5C_2 (III)	0.15	0.12	11.4	15.9
	Paramagnetic Fe	0.23	1.0	-	2.6

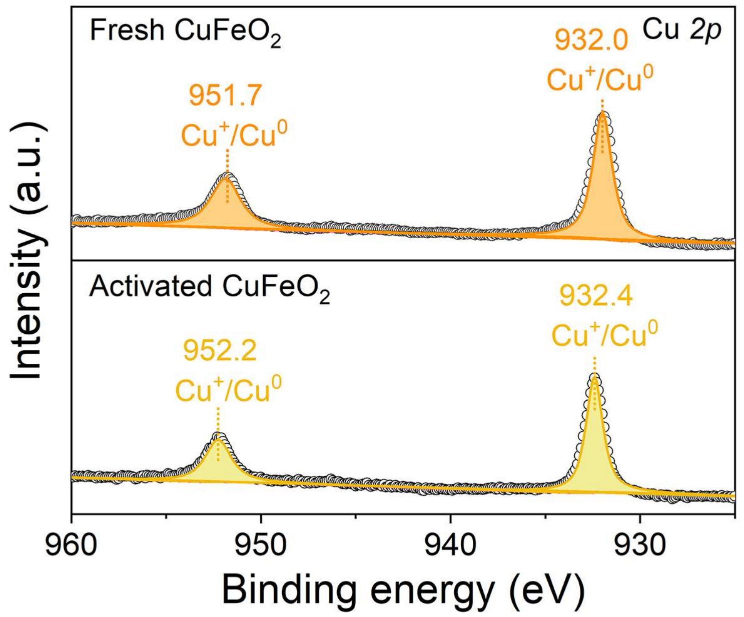

Supplementary Figure 12 | Cu 2*p* XPS spectra of fresh and activated $CuFeO_2$.

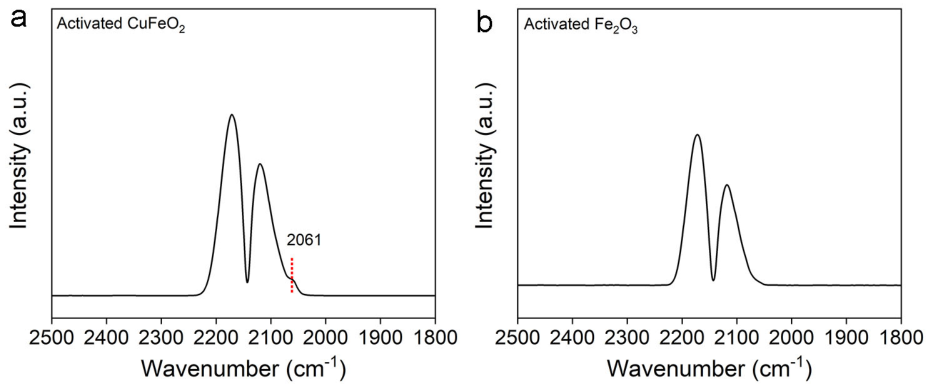

Supplementary Figure 13 | DRIFTS spectra using CO as a probe molecule. (**a**, **b**) *In-situ* DRIFTS spectra of activated $CuFeO_2$ and activated Fe_2O_3, using CO as a probe molecule. The spectra were recorded in 1 bar (CO;He = 1:9) at 25 ^oC by subtracting the background which was acquired under He flow.

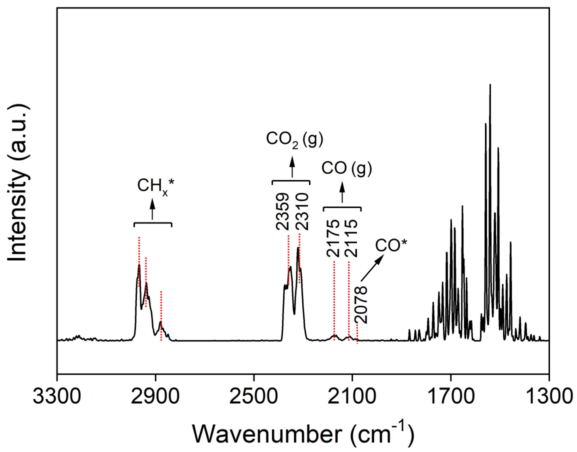

Supplementary Figure 14 | *In-situ* DRIFTS spectrum of activated $CuFeO_2$ after exposure to the mixed gas (H_2:CO_2 = 3:1, 1 bar) at 300 °C.

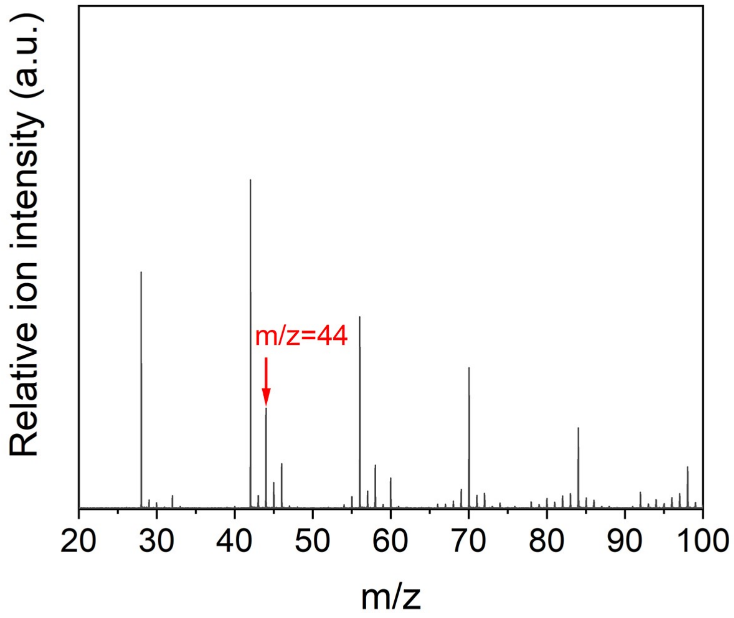

Supplementary Figure 15 | CO_2 hydrogenation over activated $CuFeO_2$ detected by SVUV-PIMS at *hν* = 10.40 eV.

Supplementary Table 6 | Ionization energy of species with m/z = 44.

Species	Ionization energy (eV)	Supplementary References
CO_2	13.77	10
C_3H_8	10.94	11
CH_3CHO	10.23	12
C_2H_4O	10.56	13
$CH_2=CHOH$	9.33	14

Analysis:

All possible species with m/z = 44 include CO_2, C_3H_8, CH_3CHO, C_2H_4O, and $CH_2=CHOH$. Their ionization energies are listed in above. Since we obtained the mass spectrum at *hν* = 10.40 eV (Fig. S8), the species with ionization energies above >10.40 eV are unable to be ionized, thereby excluding the possibilities of CO_2, C_3H_8, and C_2H_4O. Afterwards, we varied the photon energy from 9.10 to 10.40 eV. The point of inflection appeared at 10.25 eV (Fig. 4b), almost equal to the ionization energy (10.23 eV) of CH_3CHO. If $CH_2=CHOH$ existed, there would have been an inflection point around 9.33 eV. Such point was not observed in Figure 4b. Therefore, we unambiguously identified the m/z = 44 signal as CH_3CHO.

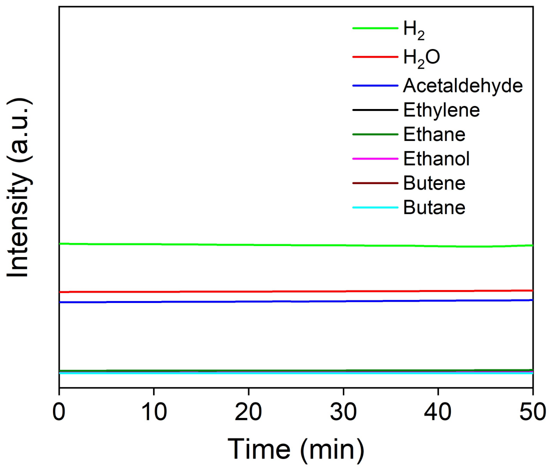

Supplementary Figure 16 | Time-dependent TPSR diagram of activated $CuFeO_2$. Note that the time was counted 10 min as soon as the temperature reached 300 °C. Then the temperature was hold at 300 °C.

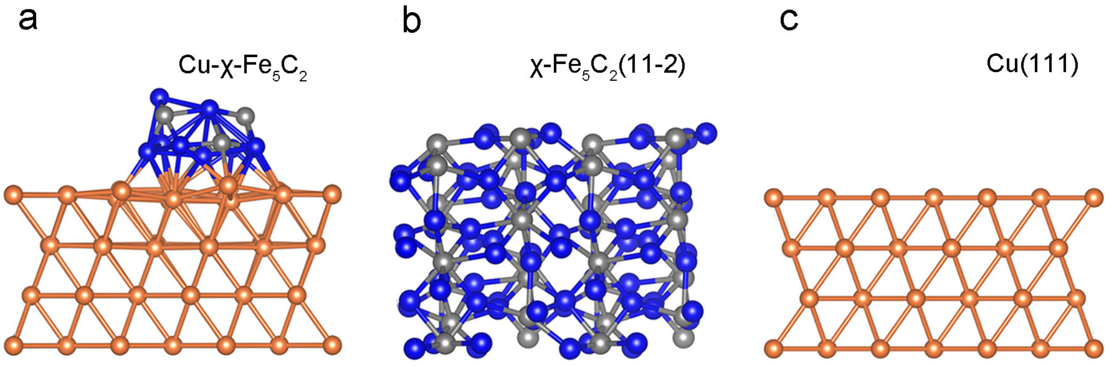

Supplementary Figure 17 | Calculated structure models. (**a**) Cu-χ-Fe_5C_2, (**b**) χ-Fe_5C_2(11-2), and (**c**) Cu(111) facets.

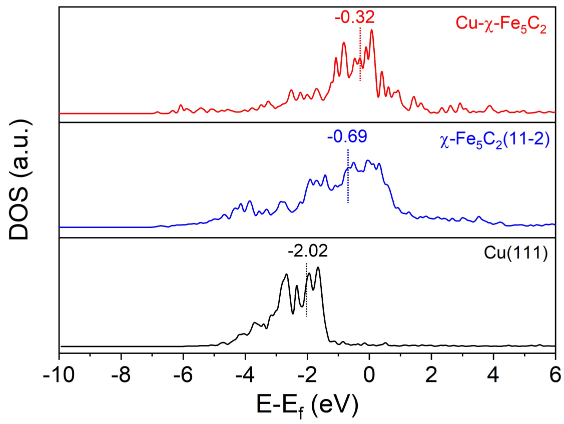

Supplementary Figure 18 | Calculated DOS and *d*-band centers of Cu-χ-Fe_5C_2, χ-Fe_5C_2(11-2), and Cu(111) facets.

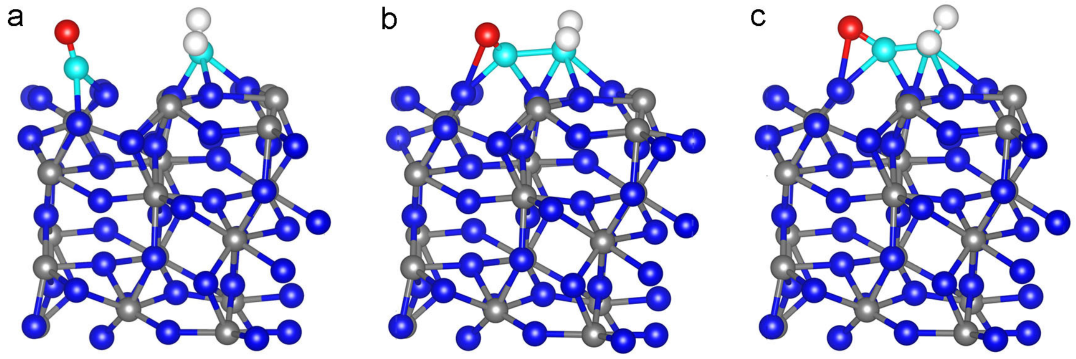

Supplementary Figure 19 | Structure models of CO insertion on χ-Fe_5C_2(11-2). Models of (**a**) CH_2 + CO, (**b**) TS1, and (**c**) CH_2CO in Figure 4c.

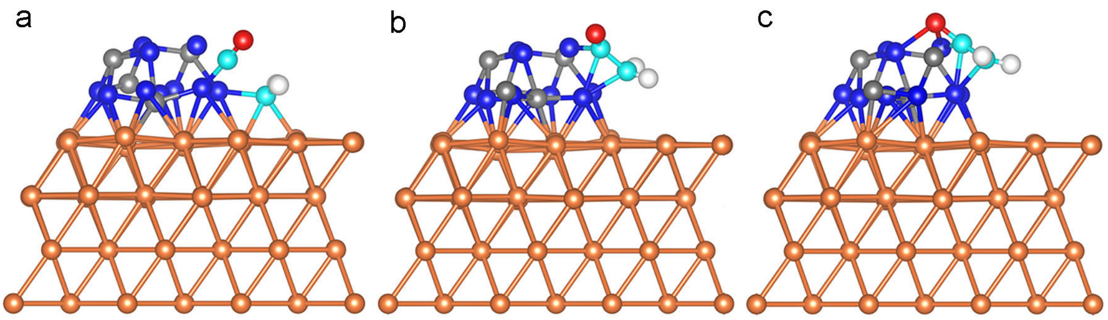

Supplementary Figure 20 | Calculated structure model of CO insertion on Cu-χ-Fe_5C_2. Models of (**a**) CH_2 + CO, (**b**) TS2, and (**c**) CH_2CO in Figure 4c.

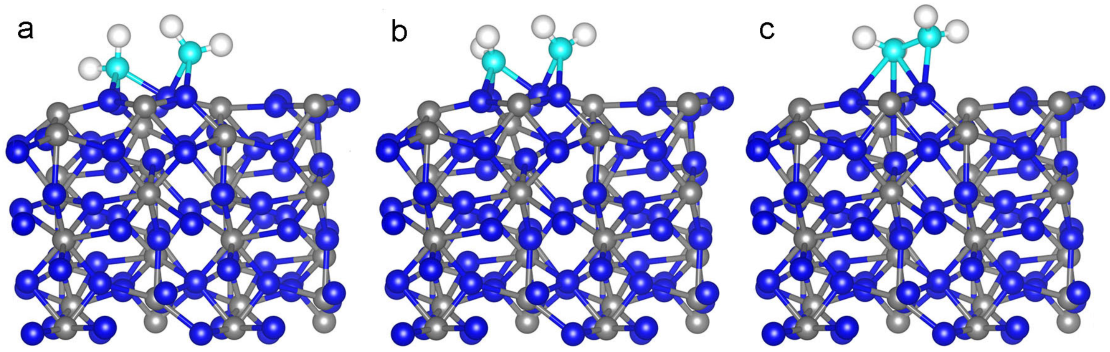

Supplementary Figure 21 | Calculated structure models of CH_2 + CH_2 coupling on χ-Fe_5C_2(11-2). Models of (**a**) CH_2 + CH_2, (**b**) TS3, and (**c**) C_2H_4 in Figure 4d.

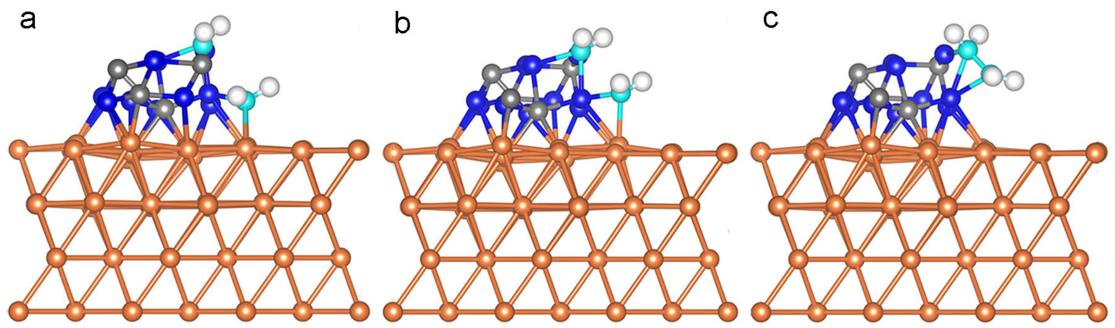

Supplementary Figure 22 | Calculated structure model of CH_2 + CH_2 coupling on Cu-χ-Fe_5C_2. Models of (**a**) CH_2 + CH_2, (**b**) TS4, and (**c**) C_2H_4 in Figure 4d.

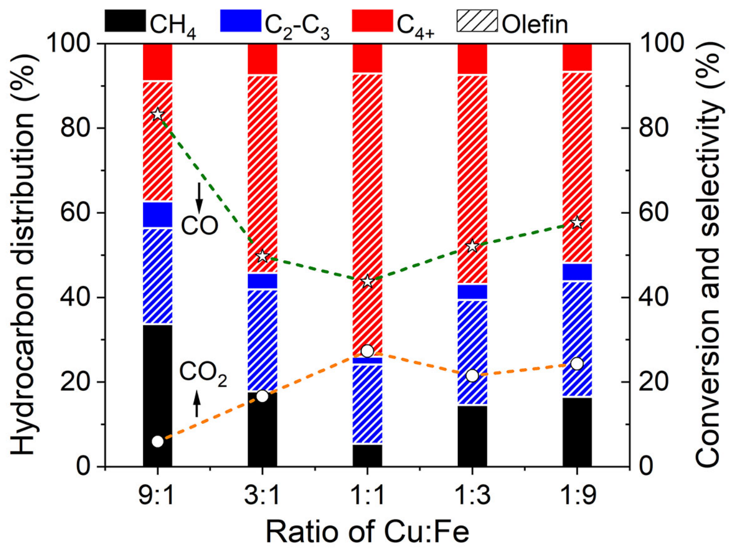

Supplementary Figure 23 | CO_2 conversion and product selectivity of activated Cu-Fe binary oxides with different Cu:Fe ratios. The reaction was conducted under 1 bar (H_2:CO_2 = 3:1) with a space velocity of 2,400 mL h^{-1} g_{cat}^{-1} at 320 °C. Time on stream = 4 h.

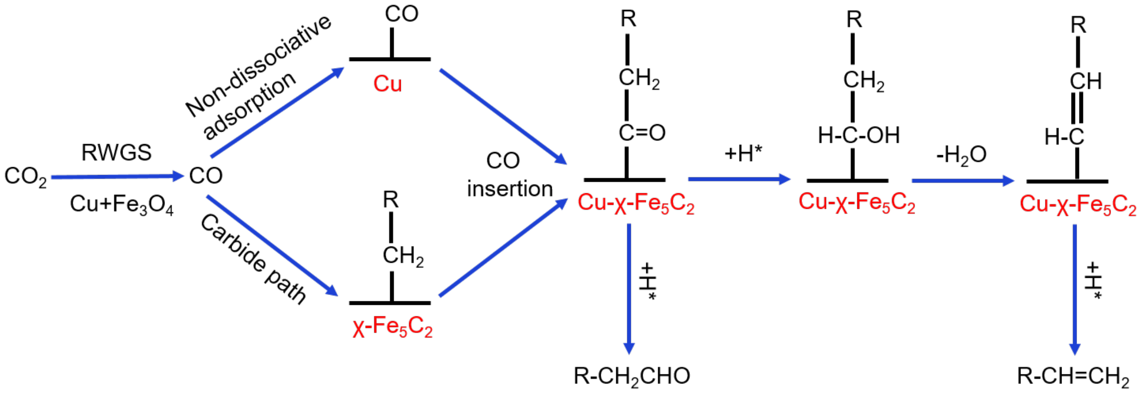

Supplementary Figure 24 | Schematic illustration of C-C coupling on the surface of activated $CuFeO_2$.

Supplementary References

1. Guo, L. *et al.* Directly converting carbon dioxide to linear α-olefins on bio-promoted catalysts. *Commun. Chem.* **1**, 11 (2018).
2. Guo, L. *et al*. Selective formation of linear-alpha olefins (LAOs) by CO_2 hydrogenation over bimetallic Fe/Co-Y catalyst. *Catal. Commun.* **130**, 105759 (2019).
3. Wang, J. *et al*. Synthesis of lower olefins by hydrogenation of carbon dioxide over supported iron catalysts. *Catal.Today* **215**, 186-193 (2013).
4. Wang, S. *et al*. Iron-potassium on single-walled carbon nanotubes as efficient catalyst for CO_2 hydrogenation to heavy olefins. *ACS Catal.* **10**, 6389-6401 (2020).
5. Kashif, K. M. *et al*. Selective conversion of carbon dioxide into liquid hydrocarbons and long-chain α-olefins over Fe-amorphous AlO_x bifunctional catalysts. *ACS Catal.* **10**, 10325-10338 (2020).
6. Visconti, C. G. *et al*. CO_2 hydrogenation to lower olefins on a high surface area K-promoted bulk Fe-catalyst. *Appl. Catal. B: Environ.* **200**, 530-542 (2017).
7. Zhang, C. *et al*. Uncovering the electronic effects of zinc on the structure of Fe_5C_2-ZnO catalysts for CO_2 hydrogenation to linear α-olefins. *Appl. Catal. B: Environ.* **295**, 120287 (2021).
8. Li, Z. *et al*. Highly selective conversion of carbon dioxide to lower olefins. *ACS Catal.* **7**, 8544-8548 (2017).
9. Choi, Y. H. *et al.* Carbon dioxide Fischer-Tropsch synthesis: a new path to carbon-neutral fuels. *Appl. Catal. B: Environ*. **202**, 605-610 (2017).
10. Shaw, D. A. *et al.* A study of the absolute photoabsorption, photoionisation and photodissociation cross sections and the photoionisation quantum efficiency of carbon dioxide from the ionisation threshold to 345 Å. *Chem. Phys.* **198**, 381-396 (1995).
11. Cool, T. A. Photoionization mass spectrometer for studies of flame chemistry with a synchrotron light source. *Rev. Sci. Instrum.* **76**, 094102 (2005).
12. Adam, T. *et al.* Determination of single photon ionization cross sections for quantitative analysis of complex organic mixtures. *Anal. Bioanal. Chem.* **389**, 1941-1951 (2007).
13. http://flame.nsrl.ustc.edu.cn/database/data.php?wid=214.
14. Cool, T. A. *et al.* Selective detection of isomers with photoionization mass spectrometry for studies of hydrocarbon flame chemistry. *J. Chem. Phys*. **119**, 8356-8365 (2003).
